# Senescence in the Development and Response to Cancer with Immunotherapy: A Double-Edged Sword

**DOI:** 10.3390/ijms21124346

**Published:** 2020-06-18

**Authors:** Anthony M. Battram, Mireia Bachiller, Beatriz Martín-Antonio

**Affiliations:** 1 Department of Hematology, Hospital Clinic, IDIBAPS, 08036 Barcelona, Spain; battram@clinic.cat (A.M.B.); mbachiller@clinic.cat (M.B.); 2 Department of Hematology, Hospital Clinic, IDIBAPS/Josep Carreras Leukaemia Research Institute, Carrer Rosselló 149-153, 08036 Barcelona, Spain

**Keywords:** senescence, SASP, senescence surveillance, inflammaging, immunotherapy

## Abstract

Cellular senescence was first described as a physiological tumor cell suppressor mechanism that leads to cell growth arrest with production of the senescence-associated secretory phenotype known as SASP. The main role of SASP in physiological conditions is to attract immune cells to clear senescent cells avoiding tumor development. However, senescence can be damage-associated and, depending on the nature of these stimuli, additional types of senescence have been described. In the context of cancer, damage-associated senescence has been described as a consequence of chemotherapy treatments that were initially thought of as a tumor suppressor mechanism. However, in certain contexts, senescence after chemotherapy can promote cancer progression, especially when immune cells become senescent and cannot clear senescent tumor cells. Moreover, aging itself leads to continuous inflammaging and immunosenescence which are responsible for rewiring immune cells to become defective in their functionality. Here, we define different types of senescence, pathways that activate them, and functions of SASP in these events. Additionally, we describe the role of senescence in cancer and its treatments, including how aging and chemotherapy contribute to senescence in tumor cells, before focusing on immune cell senescence and its role in cancer. Finally, we discuss potential therapeutic interventions to reverse cell senescence.

## 1. Defining Senescence

### 1.1. From Old to New and Different Concepts of Senescence

Cellular senescence is a cell fate that has the defining feature of stable growth arrest that is refractory to mitogenic stimulation. As such, senescent cells differ from those that are quiescent and able to reenter the cell cycle in favorable growth conditions. Senescence is distinct to apoptosis as well, and in fact, resistance to apoptosis is a feature of senescence [1]. Importantly, senescence does not correspond with a complete cellular shutdown, as evidenced by active metabolic reprogramming, changes to cell morphology and the large-scale production and secretion of proinflammatory molecules known as senescence-associated secretory phenotypes (SASP) [2]. 

**Figure 1:**
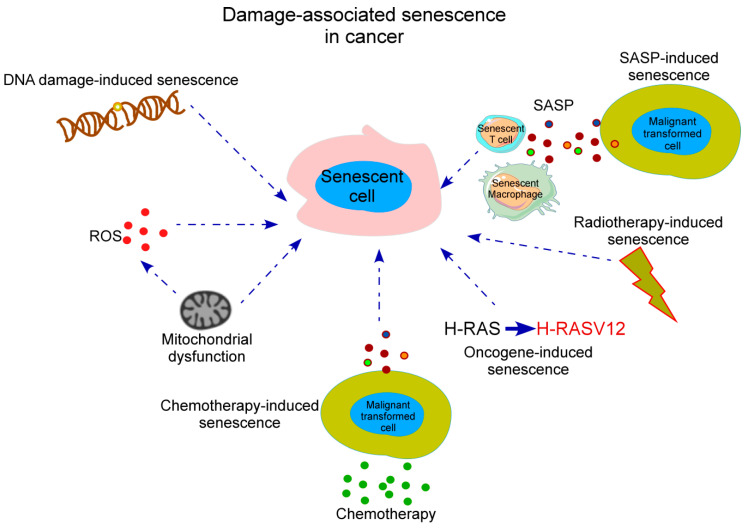
Stress-mediated inducers of cellular senescence in cancer. Damage-associated senescence associated with cancer development can occur in cells of the immune system due to SASP released by tumor cells, after accumulation of DNA damage, after inflammatory insults such as ROS or mitochondrial dysfunction which also impacts ROS production, after radiotherapy damage and chemotherapy treatments in tumor cells and also as a result of oncogenic mutations. SASP, senescence-associated secretory phenotype; ROS, reactive oxygen species.

Historically, senescence was first identified in vitro by Hayflick and Moorhead in 1961 when they observed during serial passage of human diploid fibroblasts that these cultures had limited replicative potential, becoming irreversibly arrested [3]. This impaired proliferation seemed to be associated with a progressive shortage of telomeres, which induced a persistent DNA damage response (DDR) and activated signals including p53 and p16 [4]. These events were hypothesized to be a physiological mechanism to prevent cancer initiation by avoiding proliferation of damaged cells [5] as loss of telomerase activity appeared to show inhibition of tumor progression in various mice models [6]. Since these discoveries, a high number of studies have established that, with aging, progressive accumulation of DNA damage in the cell originate a cell senescent state initiated primarily to repair this DNA damage being termed “replicative senescence” or “DNA damage-associated senescence”. Replicative senescence occurs following excessive proliferation when a cell has reached its proliferative capacity and is no longer able to undergo cell division, a point known as the Hayflick limit. Initially, damage-associated senescence causes an arrest of the cell cycle progression to potentially allow the suppression of dysfunctional, transformed, or aged cells [2,7]. Thus, senescence can be perceived initially as a tumor-protective mechanism that prevents uncontrolled replication of those precancerous cells that contain some oncogene activation or the loss of tumor suppressor genes [8]. Furthermore, it has been found that the overexpression of p53 in malignant tumors has led to induction of cell senescence and tumor regression, supporting the idea of senescence as a neoplastic brake [9]. Moreover, cellular senescence can occur prematurely after exposure to a stress signal, being termed “premature senescence”, “stress-induced senescence”, or “oncogene-induced senescence” (OIS). The extracellular or internal triggers that cause premature senescence are wide-ranging and include a high-fat diet, cytokines, radiotherapy and chemotherapy drugs (known as “therapy-induced senescence”, (TIS), reactive oxygen species (ROS), mitochondria dysfunction, and oncogene activation [10]. These stress signals can cause other cell fates, primarily apoptosis, and therefore, the balance of many different signals is critical in the cellular decision to begin a program of senescence induction (Figure 1). 

On the other hand, there is a physiological and non-damage-associated senescence termed “developmentally-programmed senescence” that occurs during mammalian embryonic development for efficient tissue remodeling. Developmentally-programmed senescence is strictly dependent on p21, followed by macrophage infiltration for clearance of senescent cells and, unlike age-related senescence, is independent of DNA damage. This mechanism has been proposed to be the origin of damage-associated senescence. Physiological cellular senescence occurring both during normal embryonic development and upon tissue damage follows a cycle of “senescence–clearance–regeneration” to trigger tissue remodeling and removal of senescent cells maintaining tissue homeostasis. This cycle is achieved with the help of the immune system that performs clearance of senescent cells. However, during aging, which is a relevant risk factor for cancer, a decline in the immune response termed “immunosenescence” causes these stages to not complete, compromising the clearance of senescent cells and exacerbating inflammation with detrimental effects [7,11]. 

### 1.2. Senescence Biomarkers and the SASP

Cellular senescence is complex and dynamic, with large variations in the observed phenotype depending on cell type, environment, and the time following senescence induction. Identification of senescent cells is dependent on multiple markers and functional properties, and no single marker has yet been identified to classify cells as being senescent. Other than the gold standard, detecting senescence-associated β-galactosidase (SA-β-gal) activity, some of the most popular senescence biomarkers are related to proliferation and the cell cycle, such as loss of Ki67 protein expression and increased levels of the cell cycle inhibitor p16. Lipofuscin, an aggregate of oxidized proteins, lipids, and metals that accumulates progressively mostly in aged postmitotic cells, colocalizes with SA-β-gal being also used as a senescence marker [12]. Other features of senescent cells include resistance to apoptosis by downregulation of caspase-3 and the proapoptotic BAX, and upregulation of antiapoptotic BCL-2, BCL-W and BCL-XL, and of cyclin-dependent kinase inhibitors p16 and p21 [13,14,15]. Genetic and epigenetic alterations can also serve as useful markers of senescence, in particular telomere length, trimethylation of histone H3 on lysine 9 (H3K9me3), phosphorylation of the histone H2AX (γH2AX), and loss of lamin B1 [16,17,18]. 

Secretion of SASP by senescent cells mediates many of their patho-physiological effects contributing to age-associated pathologies such as cancer [2,7,19]. The SASP contains a cocktail of factors that include proinflammatory cytokines, chemokines, growth factors, ROS, angiogenic factors, and proteases that are released by senescent cells [2,20] which can create a favorable cytokine microenvironment for tumorigenesis [21]. Although these proteins are secreted by many cell types in nonsenescent states, especially by tumor cells and immune cells, what makes the SASP unique is that it is induced by senescence. The exact components of the SASP are influenced by a variety of factors such as the senescence induction mechanism, cell type, and tissue of origin. Moreover, the SASP is dynamic and can change over time following the initiation of senescence [22]. The SASP is controlled by NF-κB, mammalian target of rapamycin (mTOR) [23], p38 mitogen-activated protein kinase (p38MAPK) signaling [24], and IL-1 signaling through the NLRP3 inflammasome [19]. In addition, damage-associated molecular patterns (DAMPs) can also activate the inflammasome triggering SASP activation [25]. SASP factors act in an autocrine way to create positive and negative feedback loops, and in a paracrine manner to modulate the activity of neighboring cells. Critically, the SASP can promote the propagation of senescence as it contains many prosenescent factors released in extracellular vesicles [26] which spread the SASP [19]. In the context of cancer, the SASP can have a major influence on tumor progression—it can either promote or hinder tumorigenesis depending on the exact components of the SASP [20]. In this regard, the SASP can be a double-edged sword in cancer treatment, as it is required by immune cells to mediate antitumor responses [27,28,29] promoting “senescence surveillance” and preventing tumor initiation [30,31], and at chronic levels and pathological conditions such as established tumors, SASP components, such as vascular endothelial growth factor (VEGF), CCL5, and IL-6, can induce cancer, drug resistance, cancer progression, and associated side effects such as cachexia [20,32,33,34,35,36,37,38,39,40,41]. Excellent reviews have addressed the different molecules and their functions present in the SASP [20]. Here, in Table 1 and Table 2 we have summarized some of the common molecules found in the SASP released by senescent and/or immune cells and their impact in cancer progression.

Other relevant features of senescent cells include decreased mitophagy, which results in increased mitochondria levels and defective mitochondrial networks that may contribute to the SASP and metabolic dysfunction with aging [42,43]. It is also important to highlight that senescence can be transmissible from senescent to nonsenescent cells through cytoplasmic bridges [2]. 

### 1.3. Epigenetics in Senescence

The progression of senescence is frequently associated with extensive epigenetic remodeling and large-scale chromatin reorganization [18]. Epigenetic alterations are vital for the senescence-associated DDR and p16 expression. However, epigenetic mechanisms that control senescence go much further [44]. For instance, the H3K9me3 histone modification is fundamental, and changes in the activity or levels of the enzymes associated with the deposition or removal of the methylation (methyltransferases or demethylases, respectively) can induce or reverse senescence [45,46,47]. Similarly, redistribution of histone variants is a senescence mechanism to control gene expression [44]. In particular, incorporation of histone variant H3.3 drives cell cycle arrest by downregulating proliferation-promoting genes [48], whereas, H2A.J and macroH2A1 promote the SASP [49,50]. Other important epigenetic regulators of senescence are the DNA-distorting proteins HMGB1 and HMGB2, which are diminished in the nuclei of senescent cells [51,52]. Nuclear HMGB1 inhibits SASP gene expression and HMGB2 loss is associated with the genomic reorganization that occurs early in senescence development [51,52]. Interestingly, secreted HMGB1 promotes the SASP in an autocrine/paracrine manner, in complete contrast to its role in the nucleus [52].

In addition, a common feature of OIS, but not replicative senescence, is the formation of nuclear structures known as senescence-associated heterochromatin foci (SAHFs) [16]. SAHFs occur due to reorganization of the nuclear architecture, in which areas of heterochromatin containing repressive marks, such as H3K9me3, are brought together into clusters. Formation of OIS SAHFs is dependent on the nuclear lamina component lamin B1 and DNMT1-mediated upregulation of HMGA2 [17,18]. The abundance of repressive marks and loss of modifications associated with gene expression meant that SAHFs were originally thought of as areas of gene silencing, although recent studies of OIS showing that heterochromatin decondenses as it forms SAHFs suggests that this may not be universally true [16,17]. In fact, the 3D realignment of chromatin that occurs in the formation of SAHFs actually enhances expression of senescence-associated genes in regions adjacent to SAHFs [17].

### 1.4. Signaling Pathways that Contribute to Senescence

Independent of the method of senescence induction, the DDR is often critical, but not the only cause, to translating the senescence trigger into cell cycle arrest; a key feature of senescence. In replicative senescence, telomere loss leads to the exposure of chromosome ends that the DDR detects as it would a double-strand break in DNA [53]. In OIS, hyperproliferation causes faults in DNA replication, resulting in a DDR [10]. Radiation and chemotherapeutic drugs can initiate a DDR by generating a number of different DNA aberrations, depending on the nature of the damaging agent.

Telomere attrition or irreparable/sustained DNA damage activate the kinases ataxia-telangiectasia mutated (ATM) and/or ATM and Rad3-related (ATR) to generate DDR foci (detected as γH2AX). When ATM/ATR activity at DDR foci exceeds a certain threshold, a signaling cascade is triggered in which CHK2 and CHK1, phosphorylated by ATM and ATR, respectively, translocate away from chromatin to phosphorylate a number of substrates involved in cell cycle control and protein expression [31]. As a result of this signaling, p53 is activated and stabilized, allowing p53 to drive the transcription of the cell cycle inhibitor p21, which ultimately causes cell cycle arrest at the G1/S checkpoint.

Although p21 is important for senescence initiation, it is thought that sustained cell G1 phase arrest requires a second cell cycle inhibitory protein, p16 [54]. The function of p16 is critical because it removes the inhibition of retinoblastoma protein, allowing it to repress E2F-mediated expression of DNA replication genes. Expression of p16 from the *INK4A/ARF* locus, which also encodes the tumor suppressor proteins p14 and p15, is tightly controlled by chromatin modifiers, cofactor proteins and RNA molecules [55]. Although many details of this regulation are still unknown, it is well-established that polycomb repressive complexes restrain p16 transcription by adding chromatin-compacting modifications to the *INK4A/ARF* locus, especially H3K27 trimethylation (H3K27me3). Stress signals can contribute to senescence by suppressing the polycomb repressive complexes or by activating demethylases such as JMJD3 that removes the H3K27me3 mark, both of which abolish gene silencing at the *INK4A/ARF* locus and facilitate the transcription of p16 [56,57].

A number of signaling pathways cooperate to induce the development of the SASP. The DDR and signal transduction pathways mediated by oncogene activation, p38 MAPK, cGAS/STING, and JAK/STAT ultimately converge to control the activity of NF-κB and/or C/EBPβ transcription factors. In turn, NF-κB and C/EBPβ promote the expression of SASP factors, such as IL-6, IL-8, and IL-1β, which act in an autocrine and paracrine manner to generate a positive feedback loop and increase SASP production. Moreover, SASP-derived IL-1β and TGFβ promote senescence in surrounding cells by promoting a ROS-dependent DDR [58]. mTOR signaling is key to the regulation of the SASP as well. mTOR controls the translation of key proteins involved in the SASP, such as IL-1α and MAPKAPK2 [59]. There are signaling pathways that control the flavor of the SASP as well, such as those downstream of NOTCH1 which inhibit a C/EBPβ-mediated proinflammatory SASP in favor of a TGFβ-rich secretome [60]. 

Cellular senescence is also elicited independently of the DDR. Thus, metabolic rewiring is another important contributor to the senescent phenotype, particularly in cell cycle arrest and SASP production. Senescent cells often exhibit a glycolytic state, albeit with a reduced energy profile and dysfunction in other metabolic pathways, such as the malate–aspartate shuttle [61,62,63]. Reduced malate–aspartate shuttle activity causes a decrease in the cytosolic NAD+/NADH ratio, which is critical for replicative senescence and mitochondrial dysfunction-associated senescence (MiDAS) [61,64]. The associated increase in ADP/ATP and AMP/ATP ratios trigger AMP-activated protein kinase (AMPK) activation, which promotes p53-mediated cell cycle arrest [65]. In turn, p53 causes decreased expression of the ME1 and ME2 enzymes, which convert malate into pyruvate, to further increase p53 expression and enhance senescence [66]. 

The metabolite pyruvate is another important molecule for senescence induction, although the fate of pyruvate can differ depending on the senescence trigger. In replicative senescence and MiDAS, the increase in lactate dehydrogenase activity/expression causes more pyruvate to be converted into lactate, and thus taken away from potential use in the TCA cycle [61,62]. However, in models of OIS and TIS, both glycolytic flux and TCA cycle activity are heightened [63]. Increased activity of the enzyme pyruvate dehydrogenase directs pyruvate into the TCA cycle, and as such, mitochondrial energy production is increased [67,68]. Another major driver of heightened mitochondrial metabolism in OIS is the oxidation of fatty acids [69], which are generated more in OIS cells through the action of fatty acid synthase [70]. Interestingly, OIS is sensitive to perturbation of nucleotide metabolism as well—oncogenic Ras-driven repression of a critical dNTP synthesis enzyme results in a lack of dNTP production, stalled replication forks, and, as a result, DDR [71].

The mechanisms of many other aspects of senescence, including inhibition of autophagy, morphological changes, and resistance to apoptosis, have been studied to a varied degree, but the precise cellular signal transduction pathways that control them are as yet unclear or remain controversial.

### 1.5. Contribution of Inflammaging to Senescence Surveillance

The clearance of senescent cells by immune cells known as “senescence surveillance” is a critical step for resolution of senescence. In a physiological context, the SASP promotes senescence surveillance, activating immune cells to drive the clearance of senescent cells and thus preventing tumor initiation [30,31]. With aging, the senescence of immune cells themselves, termed “immunosenescence”, avoids the elimination of senescent cells. Moreover, during aging, the SASP is responsible for the development of two different processes that lead to a chronic, sterile, highly self-reactive, systemic inflammatory condition termed “inflammaging” which enhances immunosenescence. The first process is thymic involution related to aging that leads to a decline in immune function or “immunosenescence”, causing insufficient production of naïve T cells and amplified oligo-clonal expansion of memory T cells with reduced immune repertoire diversity [72]. This immunosenescence compromises the clearance of senescent cells and exacerbates inflammation through the SASP, causing inflammaging [73]. Second, thymic involution leads to a reduced capacity of regulatory T cells (Tregs) for autoimmune suppression and to an amplified release of autoreactive T cells leading to tissue damage with chronic inflammation that exacerbates inflammaging [73,74]. 

## 2. Senescence in Cancer

One important question that still remains in the oncology field is the extent to which senescence can be placed as one of the drivers of neoplastic malignancy, as opposed to a consequence of the pathology itself. While some studies support senescence as an aftereffect willing to stop tumor growth as a proliferation-suppressive mechanism [75], others support the idea of senescence as a mechanism to create a favorable microenvironment through SASP for the tumor cells to be protected from immunoclearance [4]. In addition, it is important to highlight the dual role of immune cells in the context of cancer, as multiple lines of evidence indicate that immune inflammatory cells attracted by chemokines present in the SASP can be actively tumor-promoting, capable of fostering angiogenesis, cancer cell proliferation, and invasiveness through the secretion of proinflammatory components [76]. Thus, there is a very-heterogeneous landscape of opinions about the role of senescence in cancer. Moreover, the positive and negative aspects of cellular senescence sometimes just depend on the time senescent cells remain in the organism and whether they can be cleared by the immune system. Context-dependent outcomes and possible different scenarios regarding the relationship between senescence and cancer are discussed here.

### 2.1. Before Cancer Treatment: Age-Related Senescence and Cancer

Before cancer treatment the existence of senescence can have two different effects. On one hand, senescence can be beneficial when it is followed by immune clearance and tissue remodeling [7]. Moreover, senescence can prevent tumorigenesis by avoiding replication of precancerous cells [77]. In this context, the capacity of senescent cells entering cell cycle arrest was attributed to be a tumor-suppressive mechanism [4] and the loss of telomerase activity appeared to inhibit tumor progression in various mice models [6]. Senescence seemed to prevent uncontrolled replication of those precancerous cells that contain some oncogene activation or loss of tumor suppressor genes [8]. These observations suggested that cellular senescence does not simply arise from the accumulation of cell divisions but can also be prompted by stress inducers, such as oncogenic activation [10].

**Figure 2:**
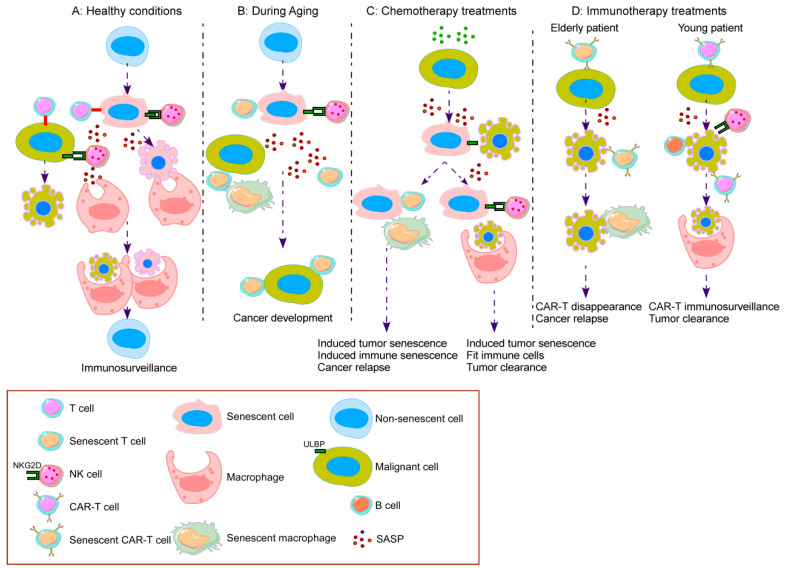
The complex and contrasting roles of senescence in the immune response to cancer. (**A**) Healthy conditions: proliferative cells react to stresses, such as DNA damage or oncogene activation, by inducing senescence to avoid malignancy. Immune cells, recruited by immunosupportive elements of the SASP, prevent the harmful effects of senescent cell accumulation by mediating their clearance in a process known as immunosurveillance. As a further protective mechanism, malignant cells are detected and eliminated by effector T cells and NK cells. (**B**) During aging: immunosenescence leads to loss of immune cell function and thus a diminished ability to detect and clear senescent cells. The SASP released from persistent senescent cells has multiple roles in cancer progression, including promoting tumor cell expansion and the development of an immunosuppressive tumor microenvironment. Cancer cells reinforce T cell senescence through metabolite competition and other mechanisms. (**C**) Chemotherapy treatments: senescence-inducing chemotherapeutic agents force malignant cells into senescence. An undesirable effect of these chemotherapies is the induction of immune cell senescence, resulting in lack of senescent cell clearance and increased risk of cancer relapse. Nonetheless, when immune cell senescence is avoided, immune cells effectively remove senescent cancer cells and tumor progression is prevented. (**D**) Immunotherapy treatments: senescence in CAR-T cells causes cell cycle arrest and loss of function, and is therefore associated with decreased in vivo persistence and cancer relapse. This problem is likely to be more pronounced in elderly cancer patients due to immunosenescence of many immune cell types. Nondysfunctional chimeric antigen receptor modified T (CAR-T) cells expand in patients to become an effective antitumor therapy. Functional NK cells, macrophages and B cells also have important roles to play in tumor clearance, making these cell types exciting future immunotherapeutic options.

On the other hand, immunosenescence is a hallmark of aging which concerns both the innate and adaptive immune system. This deterioration of the immune system contributes to the increased susceptibility to neoplastic transformation with age. Senescence can present a detrimental outcome during aging when emerging senescent cells are not cleared by the immune system leading to their accumulation. This accumulation promotes SASP and a proinflammatory state within the tissue that can cause or facilitate the appearance of neoplastic pathologies, especially in the elderly population [20]. The linkage between age, cancer, and cellular senescence remains one of the examples why senescence could end in detrimental outcomes for patients [78]. The elderly population faces different degenerative and neoplastic pathologies derived from the loss of proper cellular function. Age-related diseases, such as cancer, occur on a background of dysfunctional tissue and moreover, the appearance and accumulation of senescent cells with age could worsen the tissue status. Although the emergence of ‘primary’ senescent cells is a physiologic process of tissue maintenance for regeneration, when these cells are not efficiently cleared by the immune system due to impaired or aged immune cells, the senescent cells accumulate within the tissue [7]. Those are known as ‘secondary’ senescent cells and are generated at sites of pathology after disease initiation and are thought to be responsible for disease amplification [79] (Figure 2).

### 2.2. After Cancer Treatment with Chemotherapy

Therapy-induced senescence (TIS) appears as a result of pharmacological intervention. TIS can end either in an advantageous outcome or an unwanted side effect [79]. The most dangerous side effect derived from TIS is cancer relapse. Cancer relapse comes from dormant cancer cells that survive therapy because they become apoptosis-resistant. Anticancer agents possess the ability to induce senescent-like phenotypes in some subset of tumor cells, creating a chemo-resistant niche. Although senescent cells remain in a durable and prolonged growth arrest, it may not be permanent. In fact, studies show that while replicative senescence maintains the irreversibility of senescence [80], those caused by premature stress such as TIS or OIS can reactivate the cell cycle and bring on cancer daughter cells more transformed than the original population, suggesting that the arrested state is not indefinite. TIS cells could be contemplated as the minimal residual disease and be the origin of relapse [81]. The main hypothesis is that some senescent cancer cells generated by therapeutic intervention can eventually escape from the arrest and acquire self-renewing properties. Saleh et al. [82] showed high expression of senescence markers in cancer cell lines after they escaped TIS induced by etoposide and doxorubicin. Some detrimental features observed in those cells include aggressiveness and stemness, making them a dangerous source of therapeutic failure and cancer relapse [83] (Figure 2). 

### 2.3. After Cancer Treatment with Immunotherapy

Senescence occurrence after TIS is an explored pathway after several decades of using chemotherapeutic agents for cancer treatment. Nevertheless, more and more approaches are appearing to address anticancer treatment, such as immunotherapy [84]. Thus, the appearance of senescence after immunotherapy has not been explored as deeply as TIS. Recently, it has been identified that IFNγ and TNFα, two cytokines highly secreted after cell immunotherapy [85], induce senescence in different murine and human neoplastic diseases [86]. Moreover, we observed that natural killer (NK) cells, which are used as a source for immunotherapy [87], secrete a high variety of proinflammatory molecules, including matrix metalloproteases, heat shock proteins [88] and others present in the SASP.

## 3. Senescence in the Immune Response to Fight Cancer

The removal of senescent tumor and harmful nontumor cells by immune cells requires the inter-collaboration of different immune cell populations [9,30,89,90]. Therefore, restricting the proliferation of immune cells is likely to promote tumor growth and progression. This is particularly true for T and B lymphocytes whose function declines with aging as immunosenescence and further diminishes the natural anticancer response in older cancer patients [72]. The central hallmarks of immunosenescence include a poor immune response to novel antigens due to a decrease in naïve cells, an increase in memory cells, and also the chronic, low level of inflammation or ‘inflammaging’ with subsequent development of age-related diseases, such as cancer [73,91,92]. The cellular senescence that occurs in individual cells of the immune system is detailed below. 

### 3.1. T Cells 

T cell dysfunction occurs through the development of exhaustion, anergy, and/or senescence [93]. T cell senescence occurs naturally with aging as a result of multiple rounds of proliferation, loss of activity of the telomere-extending enzyme complex telomerase, and shortening of telomeres. As a result, this form of T cell senescence is common in older individuals but can also be found in younger people whose T cells have undergone excessive proliferation, such as in X-linked lymphoproliferative syndrome patients [94]. With aging, a thymic atrophy results in a decrease of functional naïve CD4+ and CD8 T cells [95]. This decline is more pronounced in males than in females [96] and is more noticeable for CD8 T cells with a peripheral oligo-clonal expansion of memory T cells, which in general provides a contracted T cell antigen receptor (TCR)-repertoire diversity inducing immunosenescence. In addition, aging causes CD4+ T cells to become longer-lived but functionally impaired with reduced proliferation and IL-2 production. These cells express reduced levels of the proapoptotic BCL-2 interacting mediator of cell death (Bim), which is associated with development of age-associated dysfunctions [97,98]. This T cell dysfunctionality is a common phenomenon occurring in cancer patients that leads to deficient antitumor immune responses.

Moreover, this replicative senescence is exacerbated by certain diseases, including cancer and chronic viral infections (especially cytomegalovirus (CMV)), whereby long-term exposure of antigen causes repeated T cell stimulation [99]. An alternative method of senescence induction in T cells occurs that is independent of telomere length. This premature senescence is likely to be triggered by mechanisms that provoke the DNA damage response, such as excessive ROS [100,101]. Correspondingly, metabolic competition by Tregs can promote DNA damage in effector T cells and consequently induce senescence [102,103,104]. It appears that both types of T cell senescence are immunosuppressive in the tumor microenvironment [105,106].

Senescent T cells are found within CD4+ and CD8+ T cell compartments that have lost expression of CD27 and CD28. Absence of the costimulatory molecules CD27 and CD28 in T cells corresponds with short telomeres and an upregulation of CD57 and KLRG1 [100,107,108,109,110]. Further classifying CD27-CD28- T cells with CD45RA expression identifies a CD45RA+ or T_EMRA_ population that has multiple characteristics of senescence, including decreased proliferation, an inability to upregulate telomerase activity, and higher levels of γH2AX [100,109,111]. Interestingly, these EMRA T cells do not have shorter telomeres than CD27-CD45RA- cells and the senescence phenotype is reversible, which suggests that their senescence is not purely telomere-dependent [109,112,113]. Furthermore, CD27-CD28- T cells are not resistant to apoptosis, in contrast to senescent fibroblasts, and in fact, they are more prone to activation-induced apoptosis [112,114]. Long term T cell cultures are, however, resistant to apoptosis, possibly because they are in a later stage of senescence than freshly isolated senescent T cells, or because they have been selected during the in vitro T cell expansion [115,116]. Senescent T cells also persist in vivo due to the survival signals they receive, and senescent T cells from rheumatoid arthritis patients also appear to be apoptosis-resistant, which could have implications for overall T cell responses in elderly people with cancer.

Senescent T cells lack proliferative ability and display features of cell cycle arrest at the G1/S phase transition [117]. In terms of effector function, CD27-CD45RA+ senescent T cells are very much active, retaining the ability to release inflammatory cytokines (including IFNγ and TNFα) and produce cytotoxic molecules (granzyme B and perforin) [100,110,111,114]. Interestingly, in CD4+ T cells, IL-2 production is high in CD27-CD45RA+ cells, but for CD8+ T cells, the opposite is true [113,114]. Senescent CD8+ T cells also release a cocktail of molecules that shows similarities with the SASP generated by other senescent cell types [118]. It is also important to remember that senescent T cells remain metabolically active, although senescence has a profound impact on cellular metabolism and vice versa. The ability to uptake nutrients and mitochondrial mass directly impacts on T cell senescence, as evidenced when comparing the more senescent/less proliferative CD8+ T_EMRA_ cells with less senescent/more proliferative CD8+ T_EM_ cell or CD4+ T_EMRA_ cells [100,119]. 

In many types of cancer, the presence of senescent-like T cells has been associated with malignancy and poor prognosis [120,121,122]. Indeed, tumor-infiltrating lymphocytes (TILs) have short telomeres and lack telomerase activity [123]. Although not well-researched, it has been shown that tumor cells themselves can induce a senescent-like phenotype in human T cells [120,124,125]. In a nonsuppressive environment, CD8+ TILs react to tumor antigens by proliferating, differentiating, and producing effector molecules. However, senescence causes downregulation of costimulatory molecules and, at least in some cases, effector molecules [126]. Moreover, senescent T cells repress the activity of other immune cells in the tumor microenvironment, making it even more immunosuppressive [102,103].

Recently, it has been shown that senescent T cells are increased in different hematologic malignancies, including leukemias, lymphomas, and multiple myeloma (MM) [122,127,128]. In chronic lymphocytic leukemia (CLL), patients with a CD4+:CD8+ T cell ratio below 1 had more senescent-like CD8+ T cells and a poorer prognosis [122]. A comprehensive analysis of T cells from acute myeloblastic leukemia (AML) patients showed an increase in senescent CD8+ T cells compared to healthy controls and a correlation between the expression of senescent markers and the patient response to chemotherapy [129]. Similarly, T cell senescence is associated with therapy response in MM. Specifically, when treated with autologous stem cell transplantation (ASCT), relapsed patients had drastically higher percentages of CD28-CD57+ cells in both the CD8+ and CD4+ T cell compartments compared to patients showing a complete response [130].

For solid tumors, one of the major mechanisms used by tumor cells to cause T cell dysfunction is to outcompete them for essential nutrients, especially glucose, in the tumor microenvironment [131]. Glucose deprivation induces senescence in T cells by provoking the DDR, which in turn, initiates AMPK/p38 MAPK activation [104,106,125]. In addition, tumor cells produce metabolites that induce senescence in T cells. For example, tumor cells initiate inhibitory signaling pathways in T cells by secreting adenosine, which binds to the T cell adenosine receptor, or by transferring cyclic AMP (cAMP) through gap junctions [125,132]. Another interesting senescent-promoting process that tumor cells may utilize is the release of protein- and RNA-containing exosomes, which when taken up by T cells, cause loss of CD27/CD28 expression, proliferation and IFNγ production [133]. Finally, suppressive immune cells in the tumor microenvironment promote effector T cell senescence through a number of mechanisms, such as the production of proinflammatory cytokines and nutrient competition. For instance, CD4+CD25hi Tregs and tumor-derived γδ Tregs cause DNA damage-associated senescence in effector T cells through nutrient competition, resulting in the activation of a senescence signaling network involving ATM, MAPKs (ERK1/2 and p38 MAPK), and STAT1/3 protein [103,104]. However, there are still many unknowns regarding the interplay between T cell senescence and cancer, making this area an exciting prospective area of study. 

Mechanistically, T cell senescence is regulated by signaling networks downstream of the TCR, costimulatory molecules, cytokine receptors, and inhibitory receptors. p38 MAPK is an important promoter of the senescent phenotype [100,109,134], and there are at least three pathways through which p38 MAPK can be regulated: canonical, alternative, and AMPK-mediated [134]. The canonical pathway in T cells, activated by the TCR, CD28 engagement and cytokines such as TNFα and IFNα, initiates the classical MAPK cascade [100,135,136]. The alternative pathway occurs downstream of TCR ligation whereby ZAP70 induces p38 MAPK autophosphorylation [136,137]. However, senescent T cells, which lack CD28 and TCR signalosome components, do not engage the canonical or alternative signaling cascades to regulate p38 MAPK activity [138,139]. Instead, they take advantage of a third p38 MAPK pathway in which low glucose or DNA damage, sensed by sestrins, activate AMPK, leading to TAB1-dependent autophosphorylation of p38 MAPK [138,140]. Inhibition of p38 MAPK or disruption of this pathway in senescent CD4+ or CD8+ T cells increases hTERT expression, telomerase activity, telomere length, proliferation, and TNFα secretion [100,109,138,140]. Furthermore, in senescent CD8+ T cells, p38 MAPK controls the SASP, autophagy, and mitochondrial mass [100,118], which is critical for the metabolic contribution to T cell senescence [119]. 

As well as p38 MAPK, the MAPKs ERK1/2 and Jnk also enhance T cell senescence using a similar mechanism of activation by AMPK, but without a dependence on TAB1 [140]. In addition, a recent study has discovered that CD8+ senescent-like T cells have deficient TCR activity, but adopt innate signaling mechanisms normally found in NK cells [139]. In detail, stress-sensing sestrins control this switch from TCR to NK cell signaling by regulating the expression of the NK receptor NKG2D, which then forms a complex with the adaptor molecule DAP12 to control effector molecule production and killing ability [139]. 

### 3.2. B Cells

B cells known as tumor-infiltrating B cells (TIBs) commonly permeate solid tumor microenvironments, where they can function to either inhibit or enhance tumor progression [141]. The antibodies produced by TIBs act to kill tumor cells by forming conjugates with antigens and activating complement-mediated lysis, by flagging tumor cells for destruction by other immune cells, or by stimulating the uptake of tumor cells by dendritic cells, thus activating T cells through presentation of tumor antigens [141]. In addition, TIBs act as tumor antigen-presenting cells themselves, and produce immunostimulatory cytokines and chemokines. On the other hand, B cell-derived molecules can directly enhance tumorigenesis or dampen the antitumor responses of other immune cells. As such, lymphotoxin secreted by TIBs stimulates growth in prostate cancer cells [142], and antibodies produced by B cells can form circulating immune complexes that induce tumor-resident myeloid cells to promote angiogenesis and carcinogenesis [143]. Furthermore, a subset of TIBs known as regulatory B cells suppress cytolytic immune cells through production of IL-10 and promote the formation of Tregs by secreting TGFβ [144]. Although the effect of aging on B cell tumor responses has not been studied in detail, it is tempting to hypothesize that B cell immunosenescence and cellular senescence mechanisms could dampen B cell function in cancer, particularly through the reduction of antibody-driven responses and cytokine production. 

### 3.3. NK Cells 

NK cells are important antitumor cells of our innate immune system whose activity is mediated by a complex array of activating and inhibitory receptors and their ligands on tumor cells [87]. However, NK cells are involved in many other biological processes, such as immune regulation, antimicrobial immune responses [87], and clearance of senescent cells. Therefore, a decrease in NK cell function that accompanies aging and/or cancer will have wide implications. It is important to consider the short lifespan of NK cells when talking about NK cell senescence. Estimates of the in vivo half-life of NK cells are possibly less than 10 days in humans [145], though advances in cell identification have shown a persistence of months for specific NK clones in rhesus macaque [146]. Currently, whereas for T cells there are defined phenotypes to define dysfunctional T cells differentiating exhaustion, anergy, and senescence, these phenotypes have not been clearly defined for NK cells [147]. However, given the universality of the phenomenon of senescence, NK senescence should be studied to define its existence in NK cells.

The possibility of NK cell senescence during aging has been observed with an increase in the total NK cell number with aging being the majority the CD56dim NK subset. Importantly, these NK cells show decreased cell proliferation ability [148]. Usually, NK effector cytotoxic function is defined by CD107a degranulation, IFNγ, and granzyme B production. Regarding functionality and phenotype with aging different studies have defined some changes. Specifically, from newborns to adults there is a transition from a KIR-NKG2A+ to a KIR+NKG2A- NK cell repertoire and an increase in the frequency of the senescence marker CD57 in NK cells in the elderly, which associates with human CMV infection [149]. Another study has also observed an increase in the cytotoxic NK cell subset CD16+CD56dim with greater expression of CD57, and a decrease in the immunoregulatory NK cell subset CD16-CD56bright [150]. 

In the field of cancer, there are not conclusive studies to define NK cell senescence in cancer patients. However, healthy allogeneic NK cells, such as cord-blood-derived NK cells or haploidentical NK cells are currently being used as a source of immunotherapy for the treatment of cancer patients [87]. Most groups use in vitro approaches to expand NK cells to obtain a high number of NK cells to treat patients. It could be expected that this approach, by generating high numbers of NK cells, could give rise to NK cell senescence as a result of continued in vitro proliferation. In vitro techniques to expand NK cells are able to prolong NK cell lifespan, a phenomenon observed using K562-based artificial antigen presenting cells (aAPC) with membrane-bound IL-15 or IL-21. The use of K562-based aAPC with membrane-bound IL-21 has provided superior results and interestingly, this method causes increased telomere length in NK cells [151]. However, these in vitro-expanded NK cells do not persist long in patients [152], suggesting that the enhancement of NK cell in vitro persistence and proliferation might be inducing NK cell senescence due to continuous proliferation. The phenotype of the different NK cell clones after expansion with K562-based aAPC with membrane-bound IL-21 has been investigated with no conclusive results. After 2–8 weeks of culture, NKG2A was increased and CD57 was lost. No difference in IFNγ production, NK proliferation, or NK cell cytotoxicity between CD57 positive and negative subsets was observed [153]. However, other studies, using K562 aAPC, have shown a difference evaluating the CD57 subsets and observed that CD57+ NK cells had greater IFNγ production, greater cytotoxicity, and lower proliferation after exposure to target cells than the CD57- subset [154]. Regarding SASP secretion, our group recently described that cord-blood-derived NK cells expanded with K562-based aAPC secrete a high variety of proinflammatory molecules including proteases, such as cathepsins and matrix metalloproteases [88] which are described to be part of the SASP. Further studies should be performed to find out whether these in vitro techniques to expand NK cells are actually increasing senescence in NK cells.

### 3.4. Macrophages 

Aging seems to hold an undesirable effect on macrophages as well, affecting their normal functionality. It has been observed that macrophages from old mice fail to lyse tumor cells and produce nitric oxide (NO). Moreover, aged macrophages from mice are less responsive to IFNγ, which can participate in the dysregulation of immune function in general terms [155]. Macrophages do not exhibit a stable terminally differentiated state and they represent a highly plastic population of immune cells that readjust to changing environmental circumstances. Proinflammatory cytokines such as IL-1β, IL-6, and TNFα are increased and observed in the serum of elderly individuals, suggesting the existence of inflammaging [156]. It is believed that a proinflammatory M1 macrophage phenotype prevails at tumor sites of chronic inflammation, promoting tumor survival. Once the tumor has been established, the immunosuppressor microenvironment drives macrophages towards an M2 phenotype with immunosuppressive features, which indicates a worse prognosis. In elderly mice, M2 macrophages have been found to be increased in bone marrow and spleen when compared to young mice [157]. Regarding senescent cell clearance, at all stages macrophages are a key innate immune phagocytic cell and are generally recruited by SASP molecules [158,159]. Immature myeloid cells (iMCs) characterized by CD11b+Gr1+ can differentiate into dendritic cells (DCs), neutrophils, or macrophages. However, in the presence of tumor-derived factors, they lose their capability to differentiate, supporting an immunotolerant environment [160]. Eggert et al. [39] proved that the production of CCL2 by senescent cells is able to recruit iMCs CD11b+Gr1+ cells that subsequently mature into macrophages for senescence clearance. In contrast, the absence of macrophage differentiation results in the accumulation of senescent cells [160]. 

### 3.5. Other Immune Cells

The deleterious effects of aging on immunosenescence in other immune cell types, such as DCs, invariant natural killer T (NKT) cells, and neutrophils, has been investigated [89], but very little information is available regarding the mechanism of cellular senescence that occurs in these immune cells. Interestingly, one study has reported that DCs cultured with tumor-derived γδ Tregs exhibit senescent-like features, including SA-β-gal induction and loss of function, but not the development of a SASP [103]. It is possible that mature terminally differentiated immune cells are subject to cellular senescence as well. Although unable to undergo replicative senescence as they are non-proliferative, it has been shown that terminally differentiated cells, such as neurons and hepatocytes, can exhibit signs of stress-induced senescence [161]. Intriguingly, lack of the oxidative-burst-induced DDR in neutrophils, which normally leads to apoptosis downstream of ATM signaling and ROS production, allows for the overproduction of proinflammatory cytokines, including typical SASP component IL-8, p38 MAPK activation, and repressed apoptosis—hallmarks of cellular senescence [162].

## 4. Clinical Relevance of Senescence in Immunotherapy: A Balance between Induction and Reversal of Senescence

### 4.1. Induction or Reversal of Senescence

The development of cancer treatments targeting senescence should consider both sides of senescence. Initially, the ability for a cell to induce senescence as an alternative to aberrant proliferation is a major tumor-suppressive mechanism. However, the long-term effects of senescent cancer cells are often unfavorable as SASP secretion can contribute to an enhancement of local and systemic inflammation exacerbating the side effects of cancer treatment and promoting tumor relapse and metastasis. Additionally, the senescence of noncancerous cells can aid in the generation of a procancer microenvironment and promote tumor progression. Moreover, senescent tumor cells might represent one avenue whereby they evade the cytotoxic impact of therapy, allowing for prolonged survival in a dormant state, with the potential to recover self-renewal capacity and contribute to relapse. In the case of immune cells, cellular senescence in T cells can diminish their anticancer functions and moreover, a higher number of senescent T cells prior to chemotherapy is associated with increased risk of chemotherapy-induced fatigue [82]. On the other hand, senescence may promote infiltration of immune cells to the tumor through chemokine and proinflammatory molecules secreted within the SASP [163,164]. Importantly, prosenescent therapies achieve tumor growth suspension but do not cause regression or elimination on their own, as it involves the need for a two-step therapy [165] that includes an optimal immune system capable of clearing senescent cells and eliminating residual cancerous cells [166]. As such, adoptive immune cell transfer may prove to be a useful adjuvant therapy to promote the clearance of senescent cells. 

Taken all together, combination and optimization of immune-modulatory approaches with senescence-enhancing or senolytic agents [167,168,169,170] appear as an unexplored area of research for the treatment of neoplastic diseases depending on the stage of the disease. As such, an increasing number of senolytic agents that induce apoptosis in senescent cancer cells have been studied, including dasatinib and quercetin [168] which improve clinical symptoms in pulmonary fibrosis, in aging, and after radiotherapy [171,172,173]. Others include BCL-2 family inhibitors, such as Navitoclax (ABT-263) [174]; the flavonoid fisetin [167], FOXO4-p53 interfering peptides [169], HSP90 inhibitors [170], FOXO4 D-retro reverse peptide, the mTOR inhibitor rapamycin, and antibodies targeting SASP factors such as anakinra (anti-IL-1β), tocilizumab (anti-IL6R), and infliximab (TNF-α antibody) [20].

### 4.2. Clearance of Senescent Cancer Cells by NK Cells

NK cells, which can be used in the clinic as a source of immunotherapy [87], play an important role in the removal of tumor senescent cells as demonstrated by different studies. In this regard, perforin-1 is crucial in this clearance; mice deficient in perforin-1 lack functional T, NK, or NKT lymphocytes and accumulate more senescent cells in multiple organs with signs of inflammation and overexpression of SASP. In addition, these mice exhibit premature aging in different organs and a reduced median lifespan [175]. 

NK cell receptors are involved in the NK-mediated clearance of senescent cells. NKG2D is the most studied receptor involved in these mechanisms playing a relevant role in the clearance of p53-induced senescent cells in hepatocellular cancer [176]. However, opposing roles have been demonstrated depending on the type of tumor, such as lung adenocarcinoma, where NK cells limited the clearance of senescent tumor cells and instead, infiltration of monocytes, neutrophils, and interstitial macrophages was induced [177]. Importantly, NKG2D ligands, such as MICA, MICB, and ULBP proteins, are expressed in a variety of tumor cells, which enable their targeting by NK cells [87]. Certain chemotherapies which induce senescence [2] cause over-expression of these NKG2D ligands in different types of tumors making their NK cell-mediated removal possible [178,179].

However, the role of chemotherapy in the induction of oxidative stress, SASP and senescence and in the clearance of senescent cells is controversial and can lead to opposite roles also for NK cells [4,180]. In this regard, DNA-damage chemotherapy induces secretion of SASP in different types of resistant senescent tumor cells leading to inhibition of clearance of senescent cells by NK cells. This mechanism is performed through proteases present in the SASP which cleave and release soluble NKG2D ligands [181,182,183]. These proteases include the families of matrix metalloproteinases and a disintegrin and metalloproteases (ADAMs). Among them, ADAM10 cleaves MICA, MICB, and ULBP-2 in different types of cancer cells and its increased expression correlates with cancer progression in MM [184], malignant pleural mesothelioma [185], oral squamous cell carcinoma [186], and gastric cancer [187]. Of note, inhibition of these proteases recovers immune recognition of tumor cells by NK cells [188]. On the other hand, studies demonstrated the beneficial effects of SASP components in the clearance of senescent cells, such as CCL2 secretion by senescent tumor cells in liver carcinoma which induces NKG2D ligand expression on tumor cells to activate NK-mediated clearance through NKG2D [176]. It is important to highlight that SASP drives inflammation and inflammation is recognized as one of the drivers of cancer. This association has been observed for NKG2D where in a context of exacerbated inflammation in hepatocellular carcinoma, NKG2D accelerated tumor growth [189]. Moreover, in previous stages to cancer such as hepatic fibrosis, NKG2D binding to their ligands in senescent cells induces clearance of senescent cells by NK cells, facilitating the resolution of fibrosis [190]. However, in certain cases a downregulation of NKG2D can limit these effects leading to liver fibrosis [191]. 

In advanced non-small-cell lung cancer, the chemotherapy drug pemetrexed induces senescence and increases in vitro sensitivity to NK-cell-mediated clearance of tumor cells through upregulation of ULBP proteins [192]. 

The inhibitory NK cell receptor NKG2A limits the clearance of senescent cells by NK cells. This mechanism is mediated through the SASP which induces HLA-E overexpression in senescent cells of human skin and melanocytic nevi during aging leading to NK cell inhibition [193]. 

Additional mechanisms involved in the NK-mediated clearance of senescent cells include other receptors and production of cytokines that promote macrophage activation. To give an example, in liver fibrosis, senescent cells are preferentially killed through NK cell granule exocytosis with IFNγ production protecting against liver fibrosis. In this context, granule exocytosis appears to be the preferential cytolytic mechanism instead of death receptor–death ligand interaction. The DCR2 receptor in senescent cells acts as a competitive inhibitor of death receptor signaling by death ligands, such as Fas or TRAIL, inhibiting the NK cell granule exocytosis pathway [89]. IL-15 is also involved in elimination of senescent cells after chemotherapy-associated senescence. Chemotherapy induces SASP containing IL15RA and IL-15 in MM and increased expression of IL-15/IL15RA on the membrane of senescent myeloma cells. These events allow the functional trans-presentation of IL-15 to neighboring NK cells leading to NK cell recognition and cytotoxicity [194]. Moreover, additional NK mechanisms such as antibody-dependent cellular cytotoxicity (ADCC) are activated after chemotherapy to eliminate senescent breast cancer cells, where doxorubicin enhances senescence through SASP secretion leading to a perforin-dependent increase in ADCC by NK cells [195].

The role of oxidative stress in the NK cell-mediated clearance of senescent tumor cells has been studied in breast cancer where elevated granzyme F levels and the NK cell marker NKTR correlated with recruitment of NK cells into tumors and their clearance. These activities are impaired by upregulation of the protein acyltransferase DHHC3 which supports tumor growth and metastasis. DHHC3 expression correlates with diminished patient survival in breast cancer and six other human cancer types, an effect which is mediated through a decrease in senescence and oxidative stress in tumor cells. DHHC3 ablation in mammary tumor cell xenografts increases oxidative stress promoting cellular senescence with SASP secretion that leads to recruitment of macrophages and NK cells with reduced sizes of metastasis [196]. 

The role of senolytics might be controversial for NK cells. For instance, mTOR and TNFα which are targets of senolytic drugs [20] are both required for NK cell activity. It has been shown that mTOR inhibition through peroxisome proliferator-activated receptor causes lipid accumulation in NK cells, blunting their antitumor response [197]. mTOR is also critical for IL-15 signaling in NK cells, their development, and peripheral activation [198]. Therefore, senolytic drugs targeting these pathways would potentially negatively impact NK cells and should be studied in the future. 

### 4.3. Reversal of T Cell Senescence

In the last few years, a high number of cancer immunotherapies have been developed that target CD8+ effector T cells. Currently, the most widely used immunotherapies for cancer patients are checkpoint inhibitors, specifically monoclonal antibodies against PD-1/PD-L1 and CTLA-4, that work by blocking one of the pathways that contribute to CD8+ T cell exhaustion [199]. Despite the incredible ability of these therapies to treat even late-stage cancer, 40–85% of patients treated with checkpoint inhibitors do not exhibit a sustained clinical response and many present with adverse side effects caused by autoimmune responses [200]. A novel immunotherapeutic strategy could be to reverse T cell senescence, because as explained in the previous sections, senescent T cells have an impaired ability to clear tumors and can even promote tumor progression. Indeed, senescent T cells accumulate in a multitude of different cancer types, and the increase in immune cell senescence in the elderly could be linked with their increased likelihood to develop cancer [120]. Moreover, there is evidence that treatment with checkpoint inhibitors can increase the presence of senescent T cells [201].

Among the most obvious targets to reverse T cell senescence is p38 MAPK due to its position at the heart of senescence signaling in CD4+ and CD8+ T cells. Beyond T cell senescence, impeding p38 MAPK activity is appealing for cancer treatment as a mechanism to prevent tumor cell SASP development [20]. Inhibitors that target the p38α or both the p38α and p38β MAPK isoenzymes (SCIO-469 and ralimetinib, respectively) have been used in clinical trials for myelodysplastic syndrome, MM, breast cancer, ovarian cancer, glioblastoma, and a range of advanced/metastatic cancers, albeit with mixed results [202,203,204]. Overall, the results from these trials suggest that p38α/β inhibitors are safe, and that when used in combination with chemotherapies, are promising cancer treatments. 

In T cell senescence signaling, p38 MAPK lies downstream of sestrins and AMPK [138,140], which could be other targets for senescence-reversing therapy. However, it is unclear how useful AMPK inhibitors would be in the context of an in vivo tumor as some studies found that AMPK was tumorigenic, but in other cases it acts as a tumor suppressor [205,206]. In any case, there are no specific AMPK inhibitors that are currently available [205,206]. Inhibition of sestrins, which activate AMPK, could also reduce effector T cell senescence, but could equally be detrimental because sestrins promote senescent-like CD8+ T cells to adopt an NK cell-like phenotype, which could be beneficial in tumor clearance [139].

Sestrins and AMPK regulate the ERK and Jnk MAPKs as well as p38 MAPK in senescent T cells [140]. Inhibition of MEK1/2, the MAPKKs which activate ERK1/2, prevented Treg- and tumor cell-induced responder T cell senescence in an in vivo model [102,104,125]. Three MEK1/2 inhibitors, trametinib, cobimetinib, and binimetinib, are currently approved to treat advanced metastatic melanoma where the tumor has a B-Raf V600E/K mutation, with the aim of inhibiting the constitutively active Raf/Ras/MEK/ERK pathway. As well as dampening oncogenic signaling in tumor cells, MEK1/2 inhibitors increase the number of CD4+ and CD8+ TILs without restraining their effector function [207,208,209]. In the clinic, MEK inhibitors are often used in combination with a B-Raf inhibitor, although multiple studies in different cancers have shown that they augment checkpoint inhibitor treatment as well [208,209,210,211,212], further highlighting their therapeutic potential. The extent to which MEK inhibitors control T cell senescence reversal in cancer patients, and how much that contributes to their therapeutic benefit, remains to be elucidated.

Another way to prevent T cell senescence in the tumor microenvironment is to hinder cancer cell nutrient uptake. Tumor cells are highly glycolytic and depend on a constant supply of glucose to feed their hyperactive metabolism, which in the tumor microenvironment, deprives effector T cells of glucose [131], thus promoting T cell senescence. As such, the use of drugs that obstruct glycolysis, such as glucose transporter blockers or hexokinase inhibitors, would harm tumor cells, but could also increase T cell senescence. Therefore, an interesting therapeutic approach could be to combine the use of reversible glycolysis inhibitors, which would generate a glucose-rich tumor microenvironment, with subsequent autologous T cell therapy, which would likely be more active in killing the tumor and resisting senescence. 

An alternate strategy could be to activate TLR8 signaling in tumor cells and Tregs. TLR8 agonists decrease production of cAMP in tumor cells and inhibit glycolysis in tumor-derived Tregs, but do not interfere with effector T cell metabolism, thus preventing senescence of tumor-killing TILs without limiting their function [106,125]. In a murine melanoma model, TLR8 ligands diminished the inhibitory effect of tumor cells and Tregs on effector T cells, resulting in reduced tumor size [106,125,213,214]. Clinical trials testing combination therapies containing the TLR8-specific agonist motolimod (VTX-2337) for the treatment of various tumor types have been published [215,216,217,218,219,220], and more are currently ongoing or planned (clinicaltrials.gov; NCT03906526, NCT02431559 and NCT04272333).

Finally, chimeric antigen receptor modified T cells (CAR-T cells) have appeared in recent years as a successful immunotherapy for certain hematological malignancies [84]. However, some patients treated with CAR-T cells relapse, which is partially caused by a lack of functional persistence following treatment administration [221]. As the effectiveness of CAR-T cell therapy is dependent on the fitness of the patients’ T cells from which they are derived [222], the lack of sustained functional capacity of CAR-T cells could be due to the inflated levels of T cell senescence observed in cancer patients [107]. Furthermore, many cancer patients, including those with hematological malignancies, are elderly, meaning that they are likely to exhibit immunosenescence. In the case of anti-BCMA CAR-T cell clinical trials in MM, individuals receive CAR-T cell therapy after relapse, but at this stage of disease progression, the patient T cells are highly senescent and have a phenotype associated with a diminished response to anti-BCMA CAR-T cell treatment [130,222,223]. Therefore, therapeutic interventions that reverse CAR-T cell senescence, either in the process of their development or in vivo, would be of great benefit.

### 4.4. Potential Use of B Cells in Immunotherapy and Reversal of B Cell Senescence

B cells clearly play an important role in the progression of many cancer types [141], and should therefore not be overlooked when discussing novel immunotherapies. Although some studies have shown that B cells are able to promote tumor progression and abrogate immunogenic chemotherapies [141,144], there is a clear correlation between increased TIBs and better patient outcome [141]. In fact, recent studies have shown a link between better response to immunotherapy and higher numbers of TIBs, and moreover, that this positive relationship is caused by the formation of tertiary lymphoid structures which promote T cell activation [224,225,226]. Interestingly, Helmink et al. found that there was a trend for fewer late memory CD27- IgD- B cells, which correspond to the cells that display senescent-like features, in the tumors of responders compared to nonresponders of immune checkpoint blockers [224], suggesting that fewer senescent B cells could promote immunotherapy response. 

A possible strategy to reverse B cell senescence could be to enhance autophagy. Reduced autophagy levels in old B lymphocytes corresponds with compromised B cell responses, but treatment with spermidine restores autophagy in old B cells so that they regain function [227].

### 4.5. Retuning the Tumor Microenvironment to Promote Macrophage-Mediated Cancer Cell Clearance

In the case of macrophages, the repolarization from M2 to M1 seems to be more important than senescence reversal, as M1 appears to be the phenotype capable of proinflammatory actions to initiate clearance of senescent cells [228]. The reversal of immune incompetence could be prevented by the restoration of the tumor microenvironment into one with a less immunosuppressive profile. There are studies that suggest a different role of molecules present in the SASP depending on the tumor stage [39]. Thus, in models of hepatocellular carcinoma, early stages of senescent precancerous hepatocytes secrete CCL2 via their SASP which acts as a tumor suppressive mechanism promoting the recruitment of macrophages to remove senescent cells. However, in developed tumors, senescent peritumoral tissue induces NK cell inhibition via CCL2-CCR2, promoting the growth of hepatocellular carcinoma. Moreover, CCL2 inhibits maturation of monocytes to macrophages causing more accumulation of senescent cells and contributing to tumor immune escape. This study demonstrates a dual context-dependent function of SASP components depending on the stage of the disease [39]. 

**Table 1:** SASP factors released by immune cells.

Senescent Immune Cell	SASP Factors	References
CD4+ T cells	IL-6, IL-10, TNFα, IFNγ, TGF-β1	[102]
CD8+ T cells	IL-6, IL-8, TNFα, IL-18, IFNγ, TGF-β1, CCL16, ADAM28	[102,111,117,118]
B cells	IL-6, IL-8, TNFα	[229]
NK cells	MMPs, cathepsins	[88]
Macrophages	IL-6, TNFα, PDGF-BB, TGFβ	Reviewed in [230]

IL, interleukin; TNF, tumor necrosis factor; CCL, chemokine ligand; ADAM, a disintegrin and metalloproteinase; NK, natural killer; MMP, matrix metalloproteinase; PDGF, platelet-derived growth factor; TGF, transforming growth factor.

**Table 2:** SASP factors that influence immune cell function in cancer.

SASP Factor	Senescent Cells That Secrete Factor	Protumorigenic Mechanisms	Antitumorigenic Mechanisms	Reference
IL-6	CD8+ T cells, B cells, macrophages	Recruit MDSCs, impair DC differentiation, inhibit antitumor T cell responses	Recruit macrophages and NKT cells	[231,232,233]
IL-8	Tumor cells in solid tumors and hematological malignancies	Enhancement of angiogenesis, attraction of neutrophils and MDSCs		Reviewed in [234]
IL-1α	Senescent fibroblasts. Breast cancer cells. Colon cancer cells	Regulation of IL-6 and IL-8 protumorigenic effects. Induction of production of tumor survival factors. Oncogene Ras-induced cell senescence, doxorubicin-induced cancer cell senescence and replicative senescence.	Macrophage immune surveillance	[235,236,237,238]
IL-1β	Fibroblasts	Inflammaging, induction of ROS-mediated DDR		[58]
IL-10	Macrophages	Immunosuppression		[233]
CXCL2/CXCR2	Prostate cancer cells	T cell suppression through macrophage polarization to an anti-inflammatory phenotype. Recruit iMCs that hinder tumor cell senescence.		[239,240]
PGE_2_	Hepatic stellate cells	Inhibit antitumor responses through PTGER4 receptor in hepatocellular carcinoma		[241]
CCL2	Senescent hepatocytes	Promotes accumulation of immunosuppressive iMCs promoting hepatocellular carcinoma through NK cell inhibition	Recruits myeloid cells that differentiate into macrophages to clear senescent precancerous cells	[39]
CCL3 (MIP-1 α)	Senescent hepatocytes		Recruit immune NK cells for clearance of senescent cells in hepatocellular carcinoma	[242]
TGF-β1	Fibroblasts	Inflammaging, induction of ROS-mediated DDR		[58]
TGF-β3	Senescent adipose-derived mesenchymal stem cells		Decreased angiogenic potential	[243]
CCL5	Melanoma cells		Recruit TILs to eliminate cancer cells	[244]
TNFα		Induce T cell senescence		[109]
IFNγ	Bone marrow-derived macrophages		Induce M1 macrophage differentiation	[245]
IFNα		Induce CD8+ T cell senescence, accelerates loss of CD27 and CD28		[135]

MDSC, myeloid-derived suppressor cell; NKT, natural killer T; TIL, tumor-infiltrating lymphocyte; iMC, Gr1^+^CD11b^+^ immature myeloid cell; DC, dendritic cell; IL, interleukin; PGE_2_, prostaglandin E2; TNF, tumor necrosis factor; IFN, interferon; ROS, reactive oxygen species; DDR, DNA damage response; CXCL, CXC-chemokine ligand; CXCR, CXC chemokine receptor; CCL, chemokine ligand; MIP; macrophage inflammatory protein; TGF, transforming growth factor; NK, natural killer

## 5. Conclusions

To summarize, in recent years, senescence is attracting increased attention in the field of cancer treatment as new studies are revealing its complex roles in cancer prevention, development, and progression. Whereas originally, in physiological conditions, senescence was described as a tumor suppressor mechanism, additional studies showed that damage-associated senescence after chemotherapy treatment can both hinder and promote cancer progression. The SASP has a crucial role in these seemingly contradictory outcomes and in the promotion of senescence in cells of the immune system that are crucial for the removal of tumor cells. Immunotherapy requires fit immune cells in order to succeed, and the development of novel cancer therapies should consider treatments that do not negatively affect immune cells, and moreover, that the achievement of senescence reversal in immune cells is likely to provide effective immunotherapy treatments.
